# Clinical outcomes of conversion surgery following immune checkpoint inhibitors and chemotherapy in stage IV gastric cancer

**DOI:** 10.1097/JS9.0000000000000738

**Published:** 2023-09-14

**Authors:** Huayuan Liang, Xiao Yan, Zhiwei Li, Xinhua Chen, Yaopeng Qiu, Fengping Li, Minghao Wang, Zhicheng Huang, Kaihua Huang, Qing Xie, Huimin Zhang, Rou Zhong, Zhuoyang Zhao, Yuan Zou, Jiang Yu, Yanfeng Hu, Hao Liu, Guoxin Li, Liying Zhao

**Affiliations:** a Department of General Surgery and Guangdong Provincial Key Laboratory of Precision Medicine for Gastrointestinal Tumor, Nanfang Hospital, The First School of Clinical Medicine, Southern Medical University; b Multidisciplinary Team of Gastric Tumor , Nanfang Hospital, Southern Medical University; c Department of Pathology, Nanfang Hospital, The First School of Clinical Medicine, Southern Medical University, Guangzhou, Guangdong, China

**Keywords:** chemotherapy, conversion surgery, immune checkpoint inhibitor, immunotherapy, stage IV gastric cancer

## Abstract

**Background:**

The clinical benefit of conversion surgery following immunochemotherapy in patients with stage IV gastric cancer (GC) remains uncertain. This study aims to clarify the clinical outcomes of conversion surgery for such patients.

**Methods:**

This retrospective cohort study enroled consecutive patients with stage IV GC treated with a combination of immune checkpoint inhibitors and chemotherapy and/or anti-human epidermal growth factor receptor-2 targeted therapy as first-line therapy. Cumulative survival curves were estimated using Kaplan–Meier method. Logistic regression and Cox regression analyses were conducted to identify factors associated with conversion surgery and survival, respectively.

**Results:**

Among the 136 patients included in the study. The disease control rate was 72.1% (98/136), with objective response rate in 58.8% (80/136) and complete response rate in 5.9% (8/136). Among 98 patients with disease control, 56 patients underwent palliative immunochemotherapy with median progression-free survival (PFS) and overall survival at 9.2 and 16.2 months, respectively; the remaining 42 patients underwent conversion surgery, yielding an unreached median PFS over a 19.0-month median follow-up, accompanied by 1-year overall survival and PFS rates of 96.6% and 89.1%, respectively. The R0 resection rate reached 90.5% (38/42). 7 out of 42 patients achieved pathological complete response, of whom three patients demonstrated human epidermal growth factor receptor-2 positivity. No serious complications leading to death were observed during the perioperative period. Multivariate analysis indicated that programmed death ligand 1 combined positive score greater than or equal to 5 (odds ratio, 0.22; 95% CI, 0.08–0.57; *P*=0.002) favored successful conversion surgery, while signet ring cell carcinoma (hazard ratio, 6.29; 95% CI, 1.56–25.36; *P*=0.010) was the poor prognostic factor associated with survival in patients who underwent conversion surgery.

**Conclusions:**

Conversion surgery holds the potential for significant survival benefits in stage IV GC patients who have achieved a favourable clinical response to immunochemotherapy. Individuals with signet ring cell carcinoma may experience increased post-conversion surgery recurrence.

## Introduction

Highlights

- Conversion surgery holds the potential for significant survival benefits in stage IV gastric cancer patients in the era of immunotherapy.
- The combination of immunochemotherapy and anti-human epidermal growth factor receptor-2 targeted therapy appears to yield a favourable prognosis for patients undergoing conversion therapy.
- Programmed death ligand 1 combined positive score greater than or equal to 5 was a favourable factor for successful conversion surgery, whereas individuals with signet ring cell carcinoma may experience increased post-conversion surgery recurrence.
- Significant difference between imaging and pathological staging of tumour after immunochemotherapy.

Gastric cancer (GC) patients treated with chemotherapy alone typically have a limited median overall survival (OS) of 11.1–16.0 months^1–3^. According to the REGATTA trial, gastrectomy followed by chemotherapy in stomach or gastroesophageal junction carcinoma patients did not provide additional survival benefits compared to chemotherapy alone^4^. Thus, palliative surgery combined with postoperative systemic chemotherapy is inadequate for the survival of patients with stage IV GC. Conversion therapy aims to extend patient survival by identifying patients who have achieved a favourable clinical response to chemotherapy and subsequently undergoing conversion surgery^5^.

A series of respective studies have been conducted to explore conversion therapy and the results showed that the most common regimens before conversion surgery were doublet or triplet chemotherapies based on 5-fluorouracil, platinum, and/or paclitaxel. Existing systematic chemotherapy regimens for conversion therapy have not yielded satisfactory results in terms of both conversion surgery rate, R0 resection rate, and long-tern survival^6–8^. The CheckMate-649 trial and ORIENT-16 trial have demonstrated encouraging survival of immunochemotherapy for patients with human epidermal growth factor receptor-2 (HER2)-negative advanced GC^9,10^. Moreover, the KEYNOTE-811 trial confirmed that the combination of immunochemotherapy with HER2-targeted therapy could significantly improve objective response rate (ORR) in HER2-positive GC^11^. Immunochemotherapy could be a promising direction for integrating into conversion therapy that offers a chance of radical cure to these patients. Due to the highly heterogeneous biological characteristics of stage IV GC, the optimal treatment strategy must be selected based on the individual metastasis pattern and relevant tumour biomarkers. At present, conversion therapy still faces many challenges, such as population screening, optimal surgical timing, and selection of the scope of excision^12^. The clinical benefits of subsequent conversion surgery in stage IV GC patients who respond well to immunotherapy remain uncertain.

The aim of our study was to investigate the effectiveness and safety of conversion surgery following immunochemotherapy for stage IV GC patients. The primary end points included ORR, R0 resection rate, pathological complete response (pCR) rate, progression-free survival (PFS), and OS.

## Patients and methods

### Patients

From a prospectively maintained database, we conducted a retrospective cohort study to identify consecutive enrolment patients who received first-line immunochemotherapy combined with or without targeted therapy with an initial clinical diagnosis of stage IV GC between November 2019 and August 2022. The crucial eligibility criteria were as follows: (1) histologic confirmation of GC; (2) radiographic or laparoscopic evaluation indicating unresectable stage IV disease (AJCC/AUCC 8th staging system); (3) the presence of measurable lesions meeting the Response Evaluation Criteria in Solid Tumors version 1.1 guidelines (RECIST 1.1). Exclusion criteria: (1) previous systemic treatment for GC (including targeted therapy, chemotherapy, palliative gastrectomy etc.); (2) cancer of gastric remnants and recurrence; (3) prior or co-occurrence of other malignancies. In total, 136 patients were enroled in the cohort (eFigure 1, Supplemental Digital Content 2, http://links.lww.com/JS9/B15). This study was approved by our centre’s Institutional Review Board. In addition, this study was registered at Research Registry and reported in line with the STROCSS criteria^13^, Supplemental Digital Content 1, http://links.lww.com/JS9/B14. All procedures were conducted in accordance with the ethical standards of the respective committees on human experimentation (institutional and national) and with the Helsinki Declaration of 1964 and later versions^14^.

### Biomarker assessments

Informed consent forms were signed by all patients treated with immune checkpoint inhibitors (ICIs). Programmed Death Ligand 1 (PD-L1), HER2, and mismatch repair (MMR) status were examined for immunohistochemical (IHC) staining using formalin-fixed paraffin-embedded tissue samples collected from archived tissue samples if available. PD-L1 levels were measured using the PharmDx immunohistochemistry assay (PD-L1 IHC 22C3) and represented as the combined positive score (CPS), which multiplied the number of PD-L1 stained cells (TC, lymphocytes, and macrophages) by 100. HER2 protein overexpression was evaluated using the ToGA trial scoring system based on membranous reactivity in tumour cells: 0, no reactivity or membranous reactivity in less than 10% of tumour cells; 1+, faint or barely perceptible membranous reactivity in at least 10% of tumour cells; 2+, weak to moderate complete, basolateral or lateral membranous reactivity in at least 10% of tumour cells; and 3+, strong complete, basolateral or lateral membranous reactivity in at least 10% of tumour cells^2^. HER2 positivity was defined as IHC 3+ or IHC 2+ with HER2 DNA amplification by in situ hybridization (ISH). MMR status was assessed by IHC using monoclonal antibodies for MLH1, MSH2, PMS2, and MSH6. Tumours lacking expression of MLH1, MSH2, PMS2, or MSH6 were considered MMR deficient (dMMR), while tumours maintaining expression of all four markers were considered MMR proficient (pMMR). Chromogenic in situ hybridization for epstein-barr virus-encoded RNA (EBER) was performed to assess epstein-barr virus status using fluorescein-labelled oligonucleotide probes (ZSGB-BIO, ISH-7001).

### Treatment regimens and conversion surgery

The ICIs regimens included Pembrolizumab, Sintilimab, Toripalimab, and Nivolumab. The chemotherapy regimens included Oxaliplatin plus Calcium Levofolinate, 5-Fluorouracil (FOLFOX), Oxaliplatin plus Capecitabine (CapeOX), 5-Fluorouracil Plus Leucovorin, Oxaliplatin, and Docetaxel (FLOT). Trastuzumab was recommended for patients with HER2-positive cancers. Clinicians determined the appropriate dose and treatment schedule.

Following each set of 2–4 cycles, response evaluations were carried out using computed tomography (CT). PET, or MRI were used selectively. Further radionuclide bone scintigraphy and/18F-fluoro-2-deoxy-D-glucose-PET/CT were conducted if clinically indicated. Additionally, laparoscopic staging and peritoneal cytology were also performed when necessary to detect micrometastases within the peritoneum or other areas of the abdomen. When the post-treatment efficacy evaluation reached partial response or stable disease, if preoperative imaging evaluation, staging laparoscopy, and a multidisciplinary team discussion indicated the possibility of R0 resection of tumour, surgery would be performed after obtaining patients’ informed consent, referred to our centre as conversion surgery. Conversion surgery can therefore be defined as surgical treatment of tumour initially not amenable to radical resection, with the goal of R0 resection in cases of particularly well-responding drugs. For patients with initial diagnosis of peritoneal metastases or peritoneal cytology positive, the presence of measurable lesions at the metastatic site or lymph node metastases was required, otherwise they were excluded from the study. Moreover, for such patients, conversion surgery was performed only after a second laparoscopic exploration showed no peritoneal metastasis, positive cytology, and other unresectable factors. In cases where non-curative conditions were identified, the most suitable antitumor regimen would be determined by a multidisciplinary team, considering the patient’s tolerance and response to therapy.

In downstaged patients who subsequently underwent conversion surgery, gastrectomy with more than D2 lymph nodes (LNs) were performed. Technically resectable residual metastases, such as those of the liver, ovary, and other organs, were excised simultaneously. The reconstruction modalities included Billroth II and Roux-en-Y.

### Assessment and follow-up

Treatment responses were evaluated using RECIST 1.1, and adverse events were graded according to the National Cancer Institute Common Terminology Criteria for Adverse Events version 4.0. According to AJCC/UICC’s 8th edition TNM staging system, clinical staging (cTNM), pathological staging (pTNM), and conversion therapy pathological staging (ypTNM) were classified. R0 resection was defined as the removal of primary and metastatic tumours without any evidence of malignancy detected at the proximal, distal, or circumferential margins. In addition, no distant organ metastases were observed on radiological imaging. PFS was calculated as the time between ICIs initiation and either objective tumour progression or death. OS was defined as the time from ICIs initiation to death, or the date of the last follow-up. Disease-free survival was defined as the time from conversion surgery to either objective tumour progression or death. All patients underwent imaging evaluations every 2–3 months during the treatment period. For patients who did not experience disease progression, imaging follow-up was conducted every 3 months after treatment cessation.

### Statistical analysis

Continuous variables of clinical characteristics were compared by the independent sample, unpaired *t*-test if normally distributed or the Mann–Whitney U test if non-normally distributed. Categorical variables were analyzed using either the χ2 test or the Fisher exact test. The Kaplan–Meier method and log-rank test were utilized to estimate PFS and OS, respectively. Cox proportional hazards regression was conducted to compute the hazard ratio and logistic regression was conducted to compute the odds ratio (OR), with a 95% Cl. Variables with *P* less than 0.05 in the univariable analysis were included in the multivariable model. The Hosmer–Lemeshow test was used to evaluate logistic model’s goodness-of-fit. Statistical analyses and graphical representations were performed using SPSS v.25.0 (IBM) and R v.4.03 (R Foundation). A two-sided *P* less than 0.05 was considered statistically significant.

## Results

### Study population

From November 2019 to August 2022, 136 patients were enroled in this study {56 [41.2%] women and 80 [58.8%] men; median age, 57.5 [interquartile range (IQR), 25–80] years; mean BMI, 21.7 [IQR, 16.0–29.6] kg/m^2^}. Baseline patient demographics and molecular tumour biomarkers characteristics are outlined in Table 1. According to whether patients underwent radical conversion surgery, 42 patients were assigned to the surgery group and 94 patients to the non-surgery group. As for characteristics at baseline between groups, there were no significant difference in terms of age, sex, Eastern Cooperative Oncology Group (ECOG) performance, location and size of the primary tumour, tumour invasion, LNs metastasis, signet ring cell carcinoma, histologic differentiation, laparoscopic exploration, haematology-related tumour markers, MMR, and EBER status. Compared with the non-surgery group, the proportion of several variables [IV B stage (57.1% vs. 88.3%, *P*<0.001), liver metastasis (9.5% vs. 26.6%, *P*=0.043), peritoneal metastasis (28.6% vs. 70.2%, *P*<0.001), distant LNs metastasis ([47.6% vs. 79.8%, *P*<0.001), PD-L1 CPS < 5 (31.0% vs. 58.5%, *P*=0.003), and HER2 negative (71.4% vs. 91.5%, *P*=0.002)] were lower in the surgery group.

**Table 1:** Patients baseline characteristics.

	Overall (*N*=136)	Surgery (*N*=42)	Non-surgery (*N*=94)	*p*
Age (years)				0.956
Median [IQR]	57.5 (25–80)	61.5 (25–74)	56.0 (27–80)	
Sex, *N* (%)				0.105
Female	56 (41.2)	13 (31.0)	43 (45.7)	
Male	80 (58.8)	29 (69.0)	51 (54.3)	
BMI				0.035
Mean [IQR]	21.7 (16–29.6)	22.9 (16–29.6)	21.2 (16–28.0)	
ECOG, *N* (%)				0.792
0–1	132 (97.1)	41 (97.6)	91 (96.8)	
>1	4 (2.9)	1 (2.4)	3 (3.2)	
Primary tumour size (cm), *N* (%)				0.298
<5	59 (43.4)	21 (50.0)	38 (40.4)	
≥5	77 (56.6)	21 (50.0)	56 (59.6)	
Tumour invasion, *N* (%)				0.055
cT3	4 (2.9)	3 (7.1)	1 (1.1)	
cT4a	54 (39.7)	12 (28.6)	42 (44.7)	
cT4b	78 (57.4)	27 (64.3)	51 (54.2)	
Lymph node metastasis, *N* (%)				0.149
cN1	5 (3.6)	0	5 (5.3)	
cN2	33 (24.3)	11 (26.2)	22 (23.4)	
cN3	98 (72.1)	31 (73.8)	67 (71.3)	
TNM stage, *N* (%)				<0.001
IV A	29 (21.3)	18 (42.9)	11 (11.7)	
IV B	107 (78.7)	24 (57.1)	83 (88.3)	
Location of the primary tumour, *N* (%)			0.511	
Upper	30 (22.1)	12 (28.6)	18 (19.2)	
Middle	23 (16.9)	7 (16.7)	16 (17.0)	
Lower	59 (43.4)	18 (42.8)	41 (43.6)	
Mixed	24 (17.6)	5 (11.9)	19 (20.2)	
Metastases sites, *N* (%)				
Liver				0.043
Involved	29 (21.3)	4 (9.5)	25 (26.6)	
Non-involved	107 (78.7)	38 (90.5)	69 (73.4)	
Peritoneum, *N* (%)				<0.001
Involved	78 (57.4)	12 (28.6)	66 (70.2)	
Non-involved	58 (42.6)	30 (71.4)	28 (29.8)	
Distant lymph nodes, *N* (%)				<0.001
Involved	95 (69.9)	20 (47.6)	75 (79.8)	
Non-involved	41 (30.1)	22 (52.4)	19 (20.2)	
Ovary, *N* (%)				0.745
Involved	13 (9.6)	3 (7.1)	10 (10.6)	
Non-involved	123 (90.4)	39 (92.9)	84 (89.4)	
Signet ring cell carcinoma, *N* (%)				0.172
Involved	40 (29.4)	9 (21.4)	31 (33.0)	
Non-involved	96 (70.6)	33 (78.6)	63 (67.0)	
Histologic differentiation, *N* (%)				0.565
Well or moderately	26 (19.1)	11 (26.2)	15 (16.0)	
Poorly differentiated	94 (69.1)	27 (64.3)	67 (71.2)	
Unknown	16 (11.8)	4 (9.5)	12 (12.8)	
Laparoscopic exploration, *N* (%)				0.363
Involved	93 (68.4)	31 (73.8)	62 (66.0)	
Non-involved	43 (31.6)	11 (26.2)	32 (34.0)	
PD-L1, *N* (%)				0.003
<5	68 (50.0)	13 (31.0)	46 (58.5)	
≥5	68 (50.0)	29 (69.0)	39 (41.5)	
HER2 status, *N* (%)				0.002
Negative	116 (85.3)	30 (71.4)	86 (91.5)	
Positive	20 (14.7)	12 (28.6)	8 (8.5)	
MMR status, *N* (%)				0.766
pMMR	119 (87.5)	38 (90.4)	81 (86.2)	
dMMR	8 (5.9)	2 (4.8)	6 (6.4)	
Unknown	9 (6.6)	2 (4.8)	7 (7.4)	
EBER status, *N* (%)				0.140
Negative	117 (86.0)	39 (92.8)	78 (83.0)	
Positive	7 (5.2)	2 (4.8)	5 (5.3)	
Unknown	12 (8.8)	1 (2.4)	11 (11.7)	
CEA (ng/ml), *N* (%)				0.253
<5	91 (66.9)	31 (73.8)	60 (63.8)	
≥5	45 (33.1)	11 (26.2)	34 (36.2)	
CA-199 (U/ml), *N* (%)				0.065
<37	99 (72.8)	35 (83.3)	64 (68.1)	
≥37	37 (27.2)	7 (16.7)	30 (31.9)	
CA-724 (U/ml), *N* (%)				0.087
<6	86 (63.2)	31 (73.8)	55 (58.5)	
≥6	50 (36.8)	11 (26.2)	39 (41.5)	

CA-199, carbohydrate antigen 199; CA-724, carbohydrate antigen 724; CEA, carcinoembryonic antigen; dMMR, proficient mismatch repair; EBER, epstein-barr virus; ECOG, eastern cooperative oncology group; HER2, human epidermal growth factor receptor-2; IQR, interquartile range; MMR, mismatch repair; PD-L1, programmed cell death ligand 1; pMMR, deficient mismatch repair.

### Clinical response, adverse events, and survival outcomes

As shown in Table 2, of 136 patients enroled, 80 (58.8%) patients achieved the ORR and 98 (72.1%) patients achieved the disease control rate, 38 (27.9%) patients were observed with progression at their initial response evaluation. A total of 56 (41.1%) patients suffered grade 3/4 treatment-related adverse events (TRAEs), the majority of which were myelosuppression, with anaemia being the most common [30 (22.2%) patients]. When compared to the non-surgery group, the surgery group had significantly higher rates of complete response (CR) and partial response (PR) in clinical response outcomes, but no significant differences in TRAEs were detected.

**Table 2:** Results of conversion cases in comparison to non-surgery cases regarding the clinical response and adverse events.

	Overall (*N*=136), *N* (%)	Surgery (*N*=42), *N* (%)	Non-surgery (*N*=94), *N* (%)	*p*
Clinical response				<0.001
CR	8 (5.9)	8 (19.0)	0	
PR	72 (52.9)	32 (76.2)	40 (42.6)	
SD	18 (13.2)	2 (4.8)	16 (17.0)	
PD	38 (27.9)	0	38 (40.4)	
CR response				<0.001
CR	8 (5.9)	8 (19.0)	0	
Non-CR	128 (94.1)	34 (81.0)	94 (100)	
Binary response				<0.001
Responder (CR + PR)	80 (58.8)	40 (95.2)	40 (42.6)	
Nonresponder (SD + PD)	56 (41.2)	2 (4.8)	54 (57.4)	
DCR response				<0.001
DCR	98 (72.1)	42 (100.0)	58 (59.6)	
Non-DCR	38 (27.9)	0	38 (40.4)	
TRAEs (grade 3/4)	56 (41.1)	20 (47.6)	36 (38.3)	0.308
Leucopenia	11 (8.1)	3 (7.1)	8 (8.5)	0.787
Neutropenia	11 (8.1)	4 (9.5)	7 (7.4)	0.681
Thrombocytopenia	13 (9.6)	4 (9.5)	9 (9.6)	0.993
Anaemia	30 (22.1)	11 (26.2)	19 (20.2)	0.437
Anorexia	3 (2.2)	1 (2.4)	2 (2.1)	0.926
Nausea	3 (2.2)	0	3 (3.2)	0.242
Diarrhoea	3 (2.2)	1 (2.4)	2 (2.1)	0.926
Pruritus	1 (0.7)	1 (2.4)	0	0.133
Rash	1 (0.7)	1 (2.4)	0	0.133
Hypothyroidism	3 (2.2)	1 (2.4)	2 (2.1)	0.926
Hepatitis	3 (2.2)	1 (2.4)	2 (2.1)	0.926
Ketoacidosis	1 (0.7)	1 (2.4)	0	0.133
Neuropathy peripheral	2 (1.5)	1 (2.4)	1 (1.1)	0.555
Reactive cutaneous capillary endothelial proliferation	2 (1.5)	0	2 (2.1)	0.341

CR, complete response; DCR, disease control rate; PD, progression disease; PR, partial response; SD, stable disease; TAREs, treatment-related adverse events.

**Figure 1:**
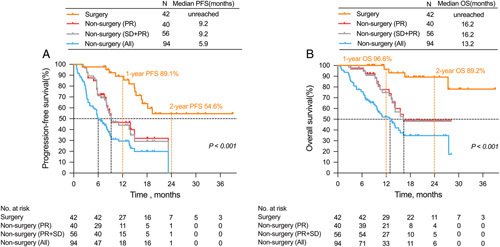
Kaplan-Meier curves for progression-free survival (PFS) between patients of conversion surgery group and non-surgery group (A). Kaplan-Meier curves for overall survival (OS) between patients of conversion surgery group and non-surgery group (B).

**Figure 2:**
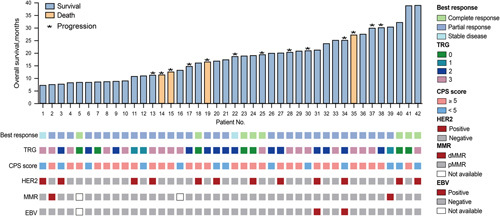
Pathological molecular characteristics and survival of patients undergoing conversion surgery.

The median follow-up time for all populations was 16.7 (IQR, 5.2–39.1) months, with the non-surgery group having a median PFS of 5.9 months and a median OS of 13.2 months. In the surgery group, the median PFS was not reached, the 1-year PFS rate was 89.1%, while the 2-year PFS rate was 54.6% (Fig. 1A). The 1-year OS rate and 2-year OS rate were 96.6% and 89.2%, respectively (Fig. 1B). The characteristics of the 13 patients who experienced recurrence after surgery are comprehensively outlined in eTable 1, Supplemental Digital Content 3, http://links.lww.com/JS9/B16, with distant LNs being the most frequent site of recurrence. Surgical patients’ initial pathological molecular features and survival are illustrated in Fig. 2. With a median follow-up time of 19.1 (IQR, 7.3–39.1) months, subgroup analysis revealed that patients who underwent R0 resection had a significantly longer median PFS than those who underwent non-R0 resection (unreached vs. 11.2 months, *P*=0.022) (eFigure 2A, Supplemental Digital Content 4, http://links.lww.com/JS9/B17). Furthermore, it was observed that patients diagnosed with signet ring cell carcinoma experienced an inferior median PFS in comparison to those without such carcinoma (unreached vs. 14.2 months, *P*<0.001) (eFigure 2B, Supplemental Digital Content 4, http://links.lww.com/JS9/B17), whereas no significant differences in the median PFS were observed between patients treated with two-drug and three-drug chemotherapy regimens (*P*=0.804) (eFigure 2C, Supplemental Digital Content 4, http://links.lww.com/JS9/B17). None of the seven patients who achieved pCR presented tumour recurrence during a median follow-up time of 19.0 (IQR, 8.5–38.9) months (eFigure 2D, Supplemental Digital Content 4, http://links.lww.com/JS9/B17). Patients with tumour regression grade (TRG) of 0–1 had a longer median PFS compared to those with TRG of 2–3 (unreached vs. 18.1 months, *P*=0.029) (eFigure 2E, Supplemental Digital Content 4, http://links.lww.com/JS9/B17). Moreover, the distribution of various chemotherapy regimens is presented in eFigure 3A, Supplemental Digital Content 5, http://links.lww.com/JS9/B18. CapeOX combined with ICIs was the most used treatment regimen. Kaplan–Meier curve analysis, excluding ICIs regimens, showed no statistically significant survival differences among the different chemotherapy regimens (*P*=0.419) (eFigure 3B, Supplemental Digital Content 5, http://links.lww.com/JS9/B18).

Simultaneously, we performed a Cox regression risk assessment model to analyze the factors associated with survival in the surgery group (eTable 2, Supplemental Digital Content 6, http://links.lww.com/JS9/B19). Univariate analysis showed that age older than or equal to 60 years and R0 resection were factors associated with favourable prognosis, while peritoneal metastasis, ovarian metastasis, and signet ring cell carcinoma were factors associated with worse prognosis. With multivariate analysis after adjustment for these significant covariates, only signet ring cell carcinoma (hazard ratio, 6.29; 95% CI, 1.56–25.36; *P*=0.010) emerged as an independent adverse prognostic factor.

### Therapeutic regimens, surgery, and pathologic results in surgery group

Table 3 presents the details of 42 patients who underwent conversion surgery. Among these patients, 31 (73.8%) received a two-drug chemotherapy regimen, while 11 (26.2%) received a three-drug chemotherapy regimen. Additionally, 12 (28.6%) patients received combination anti-HER2-targeted therapy. Notably, three patients were treated with ICIs during hospitalization after surgery without serious adverse events.

**Table 3:** Therapeutic regimens, surgical and pathological findings of patients who underwent conversion surgery.

Conversion surgery	*N*=42 Proportion, *N* (%)
Regimen	
Chemotherapeutic regimens	
Two-drug regimens	31 (73.8)
Three-drug regimens	11 (26.2)
No. cycles of induction therapy	
1–2	1 (2.4)
3–4	28 (66.6)
≥5	13 (31.0)
No. cycles of postoperative therapy	
0–4	18 (42.9)
5–8	15 (35.7)
≥9	9 (21.4)
ICIs during hospitalization after surgery	3 (7.1)
HER2 target therapy	12 (28.6)
Surgery	
Time interval to surgery (months)	1.8–32.5 (mean 4.9)
Type of gastrectomy	
Laparoscope	41 (97.6)
Open	1 (2.4)
Gastrectomy scope	
Distal	20 (52.4)
Total	22 (47.6)
Operation time (min)	104–570 (mean 325)
Amount of bleeding (ml)	20–300 (mean 74)
R0 resection	38 (90.5)
Other organs resection	
Liver	2 (4.8)
Pancreas	2 (4.8)
Ovary	1 (2.4)
Colon	1 (2.4)
Digestive tract reconstruction	
Billroth II	11 (26.2)
Roux-en-Y	31 (73.8)
Complications	
Abdominal abscess	3 (7.1)
Abdominal fluid collection	1 (2.4)
Pulmonary infection	3 (7.1)
Leakage	1 (2.4)
Postoperative pathology	
Descending stage rate (AJCC, 8 Edition)	31 (73.8)
T	33 (78.6)
N	34 (81.0)
M	15 (35.7)
ypT stage	
T0–2	16 (38.1)
T3–4	26 (61.9)
ypN stage	
N0	23 (54.8)
N1–3	19 (45.2)
ypM stage	
M0	35 (83.3)
M1	7 (16.7)
pCR	7 (16.7)
TRG	
0	7 (16.7)
1	3 (7.1)
2	14 (33.3)
3	18 (42.9)

HER2, human epidermal growth factor receptor-2; ICI, immune checkpoint inhibitor; pCR, pathologic complete response; TRG, tumour regression grade.

Postoperative pathology and radiologic imaging examination confirmed R0 resection in 38 (90.5%) patients. 7 (16.7%) patients achieved pCR, of whom three patients with HER2 positivity. 6 (14.3%) patients underwent residual metastasectomies of other organs (colon metastasis in one case, ovary metastasis in one case, pancreas metastasis in two cases, and liver metastasis in two cases). Following immunochemotherapy treatment, 9 out of 12 patients with peritoneal metastasis demonstrated disappearance of peritoneal lesions during laparoscopic examination. Pathological evaluation revealed fibrous tissue replacement in the remaining three patients who underwent peritoneal lesion resection. Among four patients with liver metastasis, two patients showed disappearance of liver metastatic lesions through laparoscopic examination; the other two underwent metastatic tumour resection, with one patient confirmed to no remaining cancer cells.

The TRG was grade 0 in 7 (16.7%) patients, grade 1 in 3 (7.1%) patients, grade 2 in 14 (33.3%) patients, and grade 3 in 18 (42.9%) patients. The most common perioperative complications were abdominal abscesses (3 patients, 7.1%) and lung infection (3 patients, 7.1%).

### Evaluation of independent risk factors for conversion surgery

Considering the significant survival benefits of patients who underwent conversion surgery at our centre, we evaluated the independent risk factors for conversion surgery in all patients by logistic regression analysis (eTable 3, Supplemental Digital Content 7, http://links.lww.com/JS9/B20). Univariate analysis showed that TNM stage IVB, presence of liver, peritoneal, and distant lymph node metastasis were risk factors for successful conversion surgery, while PD-L1 CPS greater than or equal to 5 and HER2-positive were favourable factors. Multivariate analysis including peritoneal metastasis, liver metastasis, distant lymph node metastasis, PD-L1 CPS greater than or equal to 5, and HER2-positive showed that PD-L1 CPS ≥5 (OR, 0.22; 95% CI, 0.08–0.57; *P*=0.002) was a favourable factor for successful conversion surgery, whereas peritoneal metastasis (OR, 6.73; 95% CI, 2.61–17.37; *P*<0.001) and distant lymph node metastasis (OR, 4.50; 95% CI, 1.73–11.71; *P*=0.002) were risk factors.

### Comparison between preoperative imaging combined with laparoscopic exploration staging and postoperative pathological staging

**Figure 3:**
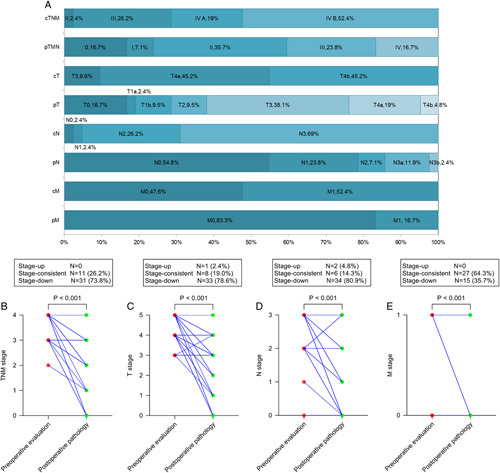
Percentage of preoperative clinical staging and postoperative pathologic staging of all patients in surgery group (A). Comparison of preoperative clinical staging with postoperative pathologic staging in TNM staging (B), T staging (C), N staging (D), and M staging (M) in surgery group.

We conducted a comparative analysis of tumour staging in 42 patients who underwent conversion surgery. Specifically, we compared the tumour staging results obtained from a combination of the last preoperative chemotherapy imaging and laparoscopic exploration with staging based on postoperative pathology evaluations. Our findings revealed noteworthy differences between the two staging methods. Regarding the total TNM stage, the rate of stage IV disease assessed by postoperative pathology was significantly lower than that assessed by preoperative examination (16.7% vs. 71.4%) (Fig. 3A). Postoperative pathology confirmed that 73.8% of patients achieved downstage, and up to 35.7% achieved downstage to stage II (Fig. 3B). In terms of the primary tumour stage (T), similarly, a lower rate of T4 was observed in postoperative pathology evaluation compared to preoperative examination (23.8% vs. 90.4%), with 78.6% of patients achieving downstage (Fig. 3C). As for the nodal stage (N), the rate of N3 was significantly lower in postoperative pathology compared to preoperative examination (14.3% vs. 69%), with 80.9% of patients achieving downstage, and up to 54.8% achieving downstage to N0 stage (Fig. 3D). For the metastasis stage (M), the rate of consistency between preoperative and postoperative staging was 64.3%, and 35.1% of patients’ M staging fell from M1 to M0 (Fig. 3E).

## Discussion

In this study, we reviewed a consecutive cohort of patients receiving immunochemotherapy and/or anti-HER2-targeted therapy as first-line therapy. For patients who have shown treatment efficacy, some of them underwent conversion surgery. Our findings highlight the potential of conversion surgery as a promising therapeutic approach for stage IV GC patients, offering significant survival benefits.

Previous retrospective studies have reported a range of R0 resection rates following post-chemotherapy conversion surgery, ranging from 56.4 to 84.8%^7,15,16^, with a median OS ranging from 22.5 to 50.0 months^8,17,18^. But these studies have not reported whether there is a significant difference in survival between non-surgical and surgical treatments for patients with stage IV GC who response well to chemotherapy. Recently, the preliminary results of the CO-STAR trial, an ongoing single-centre, single-arm trial, evaluating the efficacy of Sintilimab, Apatinib, and chemotherapy for stage Ⅳ GC conversion treatment, have been published. The results showed higher rates of response (61.7%, 29/47) and R0 resection (59.6%, 28/47), along with pathological complete response (17.2%, 5/29)^19^. However, it did not address whether conversion surgery could provide added survival benefits for surgical patients. In this study, postoperative pathological analysis revealed a favourable R0 resection rate of 90.5% (38/42) and a pCR rate of 16.7% (7/42). Notably, non-surgical patients who achieved disease control rate or ORR in terms of the best tumour response had a median PFS of 9.2 months and a median OS of 16.2 months. In contrast, surgical patients with the same clinical tumour response demonstrated a significant prolongation in median PFS and OS, with 1-year and 2-year OS rates of 96.6% and 89.2%, respectively. Our study provides evidence that conversion surgery confers substantial survival benefits to patients who have exhibited positive treatment response to immunochemotherapy.

For the choice of regimens for conversion therapy, we compared the clinical outcomes of various chemotherapy regimens in combination with ICIs. The results showed no statistical difference in survival benefit between the groups without considering regimens of ICIs. However, by analyzing the Kaplan–Meier curves, patients on the regimen of CapeOX combined with trastuzumab and ICIs had a lower relapse rate after conversion surgery (eFigure 3A, Supplemental Digital Content 5, http://links.lww.com/JS9/B18). In addition, 7 out of 42 patients achieved pCR, of whom 3 patients demonstrated HER2 positivity. After anti-HER2 treatment, in tumours exhibiting HER2 amplification, there would be a downregulation in the release of cytokines, including CCL2, CCL21, VEGF, and CXCL1, which leads to an amelioration of the immunosuppressive factors within the tumour microenvironment^20^. As confirmed by the interim results of the KEYNOTE-811 trial^11^, the combination of immunochemotherapy and anti-HER2-targeted therapy has the potential to enhance the efficacy of ICIs and appears to yield a more favourable prognosis for patients undergoing conversion therapy, but further clinical research is needed for confirmation.

Comprehensive molecular typing can provide a basis for the screening of sensitive populations for conversion therapies and the selection of effective regimens. The CheckMate-649 trial and ORIENT-16 trial have demonstrated encouraging survival of immunochemotherapy for advanced GC patients with CPS greater than or equal to 5. In this study, logistic regression analyses showed that stage IV GC patients with PD-L1 CPS greater than or equal to 5 were more likely to undergo successful conversion surgery after immunochemotherapy. Furthermore, upon conducting a subgroup analysis within the surgery group, we observed a significant association between the presence of signet ring cell carcinoma and tumour recurrence. Notably, among the nine patients who underwent R0 resection with signet ring cell carcinoma, seven experienced recurrences, with a median PFS of 14.2 months. In fact, signet ring cell carcinoma of GC has been found to be a risk factor for progression consistent with previous studies^21^. Caution should be exercised when performing conversion surgery on such patients, further research is required to investigate the heterogeneity and microenvironmental characteristics of signet ring cell carcinoma^22^.

Currently, peritoneal dissemination is often deemed oncologically unresectable with poorer survival^23^. Chinese data from the CheckMate-649 trial showed that patients with peritoneal metastasis who received immunochemotherapy had a worse median OS than those without peritoneal metastasis (9.5 vs. 15.6 months)^24^. In our study, among 12 patients with peritoneal metastasis at first visit time who underwent conversion surgery after immunochemotherapy, the median PFS was 15.0 months (eFigure 2F, Supplemental Digital Content 4, http://links.lww.com/JS9/B17), all of whom were not found peritoneal metastatic lesions during conversion surgery. Our findings suggest that for patients with peritoneal metastasis who response well to immunochemotherapy, active laparoscopic exploration should be conducted to determine the feasibility of R0 resection.

Accurate tumour staging plays a crucial role in guiding treatment decisions and predicting prognosis. Previous studies have demonstrated substantial disparities between histologic and radiologic staging following neoadjuvant therapy^25–27^. The radiologic concordance comparing baseline CT to pathologic staging ranges from 69 to 88% for T-stage and 51 to 71% for N-stage^28,29^. In our study, despite utilizing both radiologic imaging and laparoscopic exploration after immunochemotherapy to ascertain patients’ tumour staging post-treatment, the agreement between preoperative and postoperative staging was low for T (19%) and N (14.3%) stages. A prior study identified “nodal immune flare” in neoadjuvant ICI-treated patients, where radiologically abnormal nodes post-therapy lacked cancer and instead showed new non-caseating granulomas upon pathological evaluation^30^. Immunochemotherapy-induced inflammation and oedema hinder tumour invasiveness assessment, and radiologic imaging has a low ability to differentiate immunochemotherapy-induced fibrosis from vital tumour tissue, leading to overstaging of T-category and N-category^31^. For stage IV GC, accurate M staging is crucial for assessing the feasibility of R0 resection. The primary factor influencing M staging is the identification of intraperitoneal metastasis, which encompasses peritoneal metastasis and peritoneal cytology positive. Laparoscopy has demonstrated greater sensitivity in detecting peritoneal metastasis, leading to its inclusion in current guidelines as a recommended approach for advanced GC^32,33^. In the surgical group of this study, 73.8% of all patients underwent a second laparoscopic exploration to clarify intra-abdominal staging. The concordance between preoperative staging and pathologic staging was 64.3%. To our knowledge, this is the first study to compare radiologic staging and pathological staging in stage IV GC after immunochemotherapy. In the era of immunotherapy, certain patients exhibit positive responses to treatment, yet favourable response may not be evident through radiographic imaging, resulting in missing the only opportunity for R0 resection. Consequently, there is a growing need for more precise methods to evaluate tumour response to immunochemotherapy and accurately identify patients who are appropriate candidates for R0 resection.

We simultaneously evaluated the safety of conversion surgery following immunochemotherapy. GC patients receiving immunochemotherapy treatments have reported TRAEs in previous clinical researches, potentially originating from immunological causes and affecting various physiological systems such as the endocrine, gastrointestinal, liver, lung, kidney, and skin^34,35^. Chemotherapy-related complications were mostly observed in gastrointestinal and skin-based reactions^36^. Comparatively, our study showed that anaemia was most frequently observed during treatment. Surgery-related complications were minimal and safe, similar to adverse events reported during ongoing neoadjuvant chemotherapy for GC^37^. No serious complications leading to death were found during the perioperative period, which was consistent with previous studies reporting conversion surgery after chemotherapy^7,8^. Therefore, these results indicate that immunochemotherapy did not increase the incidence of perioperative adverse events.

There are still some limitations to this investigation. Firstly, this is a retrospective, single-centre study with a small sample size and a short follow-up period. Secondly, due to the biological heterogeneity of stage IV GC and individualized differences, there is considerable inconsistency in aspects such as chemotherapy regimens, extent of surgical resection, and timing of operation. Most of these decisions were made on a case-by-case basis based on multidisciplinary team discussion. Nevertheless, this study suggests that surgical intervention following immunochemotherapy may be associated with a higher rate of complete resection and potential improvements in survival.

## Conclusion

Our study suggests that conversion surgery holds the potential for significant survival benefits in stage IV GC patients who have achieved a favourable clinical response to immunochemotherapy. Individuals with signet ring cell carcinoma may experience increased post-conversion surgery recurrence. Furthermore, this study is expected to serve as a reference for subsequent large-scale clinical research on conversion therapy of GC.

## Ethical approval

The study was approved by the Institutional Review Board of Nanfang Hospital of Southern Medical University (application number: NFEC2023375).

## Consent

As this study was a retrospective study and did not include any potentially identifiable patient data, informed consent to be included in the study was not obtained from the enroled patients. The institutional review board gave the ethics approval for this retrospective study.

## Source of funding

This study was supported by grants 82172814 (L.Z.) from the National Natural Science Foundation of China; grant 2022A1515010267 (L.Z.) from the Guangdong Natural Science Fund; grant 2019JC05Y361 (G.L.) from the Guangdong Provincial Major Talents Project.

## Author contribution

H.L.: collect patient data, drafting of the manuscript, critical revision of the manuscript for important intellectual content. X.Y.: collect patient data, drafting of the manuscript. Z.L.: Collect patient data, drafting of the manuscript, statistical analysis. X.C.: collect patient data, drafting of the manuscript. Y.Q. and F.L.: Critical revision of the manuscript for important intellectual content. M.W., Z.H. and K.H.: statistical analysis. Q.X., H.Z., R.Z., Z.Z. and Y.Z.: collect patient data, methodology and validation. J.Y, Y.H, H.L.: supervision. G.L.: obtained funding, supervision. L.Z.: drafting of the manuscript, obtained funding, supervision.

## Conflicts of interest disclosure

The authors declare that the paper was conducted in the absence of any commercial or financial relationships that could be construed as a potential conflict of interest.

## Research registration unique identifying number (UIN)

1. Name of the registry: ClinicalTrials.gov.

2. Unique Identifying number or registration ID: researchregistry9204.

3. Hyperlink to your specific registration (must be publicly accessible and will be checked): https://www.researchregistry.com/browse-the-registry#home/registrationdetails/649c529114f79c0029fbc1a9/.

## Guarantor

Prof. Dr Liying Zhao MD, Department of General Surgery and Guangdong Provincial Key Laboratory of Precision Medicine for Gastrointestinal Tumor, Nanfang Hospital, The First School of Clinical Medicine, Southern Medical University, Guangzhou, Guangdong, China. Tel.: +86 13430396746, E-mail: zlyblue11@163.com.

## Provenance and peer review

Not commissioned; externally peer-reviewed.

## Data availability statement

The data that support the findings of this study are available from the corresponding author upon reasonable request.

## Supplementary Material

SUPPLEMENTARY MATERIAL
