# Supplementary material for: Clinical outcomes of conversion surgery following immune checkpoint inhibitors and chemotherapy in stage IV gastric cancer
Source: Int J Surg. 2023 Sep 14;109(12):4162–72. doi: 10.1097/JS9.0000000000000738 (PMC10720795; doi:10.1097/JS9.0000000000000738)

eFigure 1. Flow chart of patient eligibility for inclusion in the study.

Abbreviations: GC, gastric cancer; ICIs, immune checkpoint inhibitors; PD, progression disease; DCR, disease control rate.


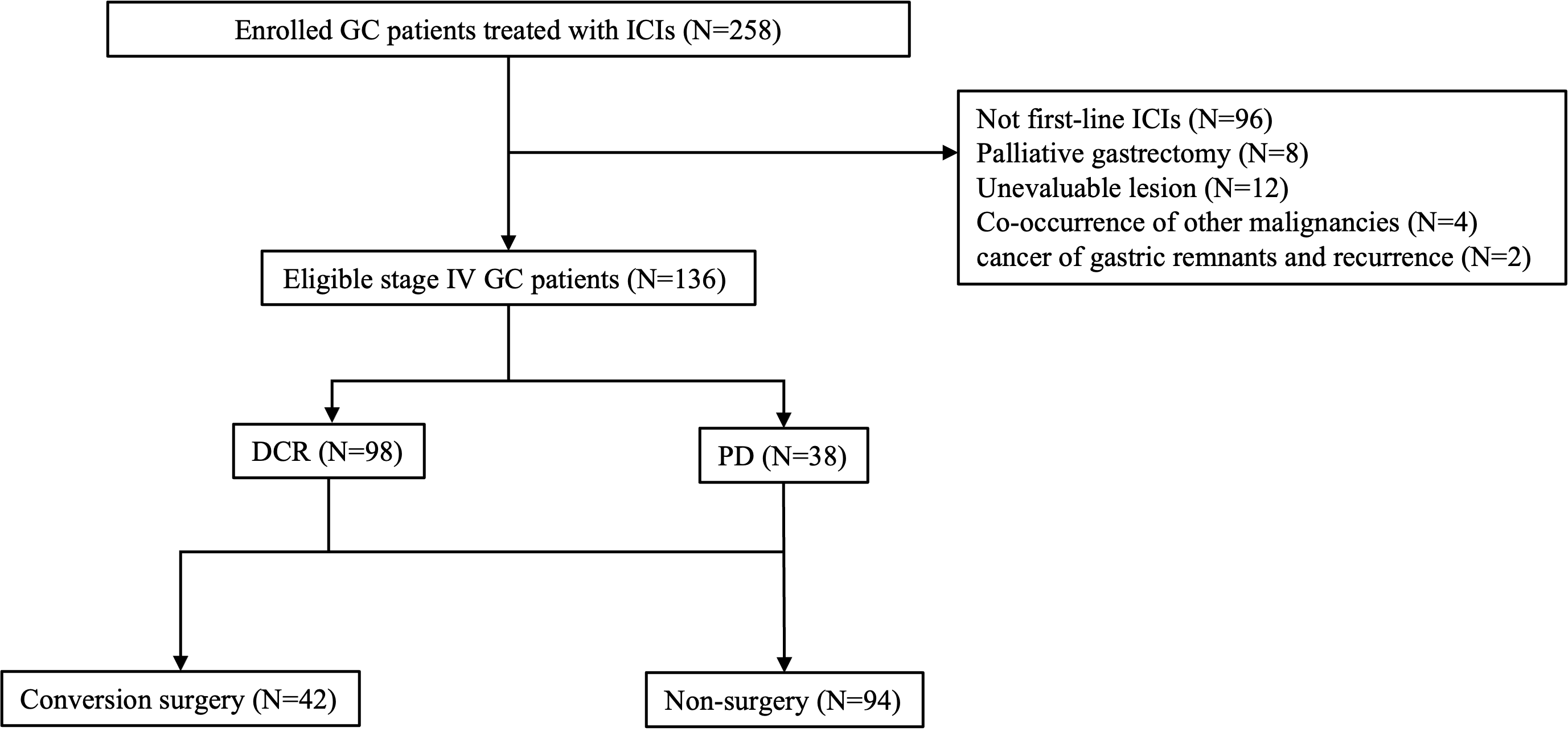

Supplement: SUPPLEMENTARY MATERIAL [file js9-109-4162-s002.docx]
