# Supplementary material for: Clinical outcomes of conversion surgery following immune checkpoint inhibitors and chemotherapy in stage IV gastric cancer
Source: Int J Surg. 2023 Sep 14;109(12):4162–72. doi: 10.1097/JS9.0000000000000738 (PMC10720795; doi:10.1097/JS9.0000000000000738)
Supplement: SUPPLEMENTARY MATERIAL [file js9-109-4162-s003.docx]

| **eTable 1. Clinicopathological data of 13 patients with recurrence after conversion surgery** | | | | | | | | |
| --- | --- | --- | --- | --- | --- | --- | --- | --- |
| No. | Sex | Age | Regimen | Clinical staging (Initial ) | Other organs resection | ypTNM | DFS (months) | Recurrence site |
| 1 | M | 42 | FOLFOX+Sintilimab | T4aN3M1 | Partial liver | T4aN3M0 | 12.4 | LNs |
| 2 | F | 31 | CapeOX+Toripalimab | T4bN3M1 | Ovary | T4bN1M1 | 8.4 | LNs, peritoneum |
| 3 | F | 25 | FOLFOX+Sintilimab | T4bN3M1 | NA | T4aN0M0 | 9.1 | Anastomosis, LNs |
| 4* | M | 69 | CapeOX+Toripalimab | T4bN3M0 | Partial liver | T3N1M1 | 5.8 | LNs, peritoneum |
| 5* | M | 44 | FLOT+Toripalimab | T4bN3M1 | Partial pancreas | T3N0M1 | 4.7 | LNs, peritoneum |
| 6 | M | 42 | CapeOX+Toripalimab | T4bN3M0 | NA | T4bN3M0 | 13.7 | LNs |
| 7 | M | 56 | FLOT+Pembrolizumab | T4bN2M0 | NA | T4aN0M0 | 14.2 | Gastric stump, LNs |
| 8 | F | 28 | FLOT+Toripalimab | T4bN2M0 | NA | T4aN1M0 | 16.6 | LNs |
| 9 | M | 57 | CapeOX+Toripalimab | T4bN3M0 | NA | T3N0M0 | 9.4 | LNs, peritoneum |
| 10 | M | 68 | CapeOX+Trastuzumab+Toripalimab | T4aN2M1 | NA | T3N0M0 | 10.9 | LNs |
| 11 | F | 50 | CapeOX+Trastuzumab+Toripalimab | T4aN3M1 | Partial colon | T0N3M1 | 6.5 | Peritoneum |
| 12 | F | 61 | FLOT+Nivolumab | T4bN2M0 | NA | T3N1M0 | 23.3 | LNs |
| 13 | F | 43 | FLOT+Toripalimab | T4bN3M1 | NA | T4bN3M0 | 11.7 | LNs |
| Abbreviations: F, female; M, male; LNs, lymph nodes; DFS, disease free survival; NA, not applicable; *Positive peritoneal lavage cytology. | | | | | | | | |
