# Supplementary material for: Clinical outcomes of conversion surgery following immune checkpoint inhibitors and chemotherapy in stage IV gastric cancer
Source: Int J Surg. 2023 Sep 14;109(12):4162–72. doi: 10.1097/JS9.0000000000000738 (PMC10720795; doi:10.1097/JS9.0000000000000738)

eFigure 3A. The proportion of chemotherapy regimens among patients in surgery subgroups.

eFigure 3B. Kaplan-meier curve for progression-free survival (PFS) among patients with different chemotherapy regiments in surgery subgroups.


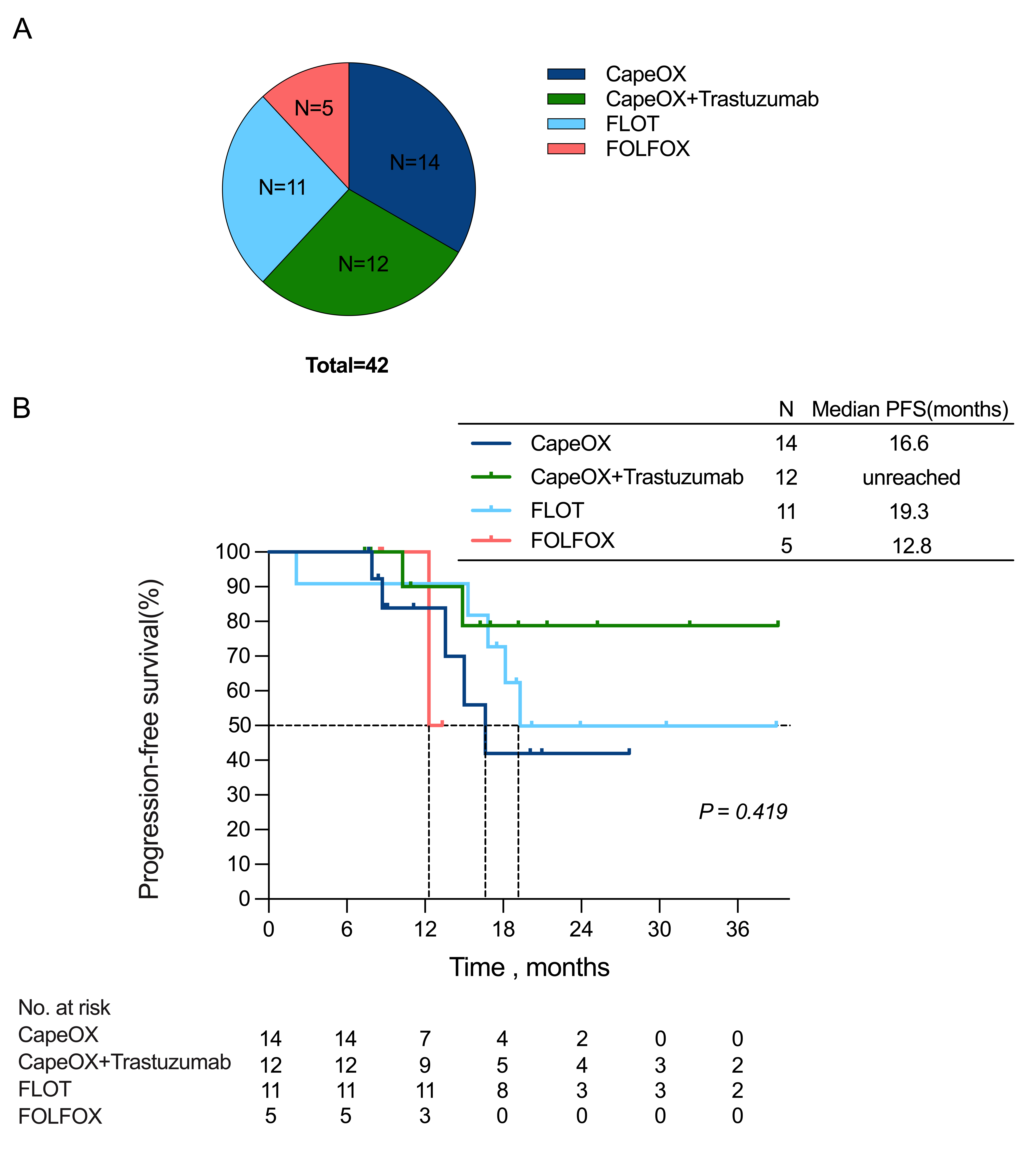

Supplement: SUPPLEMENTARY MATERIAL [file js9-109-4162-s005.docx]
