# Supplementary material for: Clinical outcomes of conversion surgery following immune checkpoint inhibitors and chemotherapy in stage IV gastric cancer
Source: Int J Surg. 2023 Sep 14;109(12):4162–72. doi: 10.1097/JS9.0000000000000738 (PMC10720795; doi:10.1097/JS9.0000000000000738)
Supplement: SUPPLEMENTARY MATERIAL [file js9-109-4162-s006.docx]

| **eTable 2. PFS and association with clinicopathologic characteristic using Cox regression of conversion surgery cases** | | | | | |
| --- | --- | --- | --- | --- | --- |
|  | Univariable analysis | |  | Multivariable analysis | |
| Clinicopathologic variable | HR (95% CI) | P value | | HR (95% CI) | P value |
| Female vs male | 1.85 (0.62-5.52) | 0.269 | |  |  |
| Age at diagnosis |  |  | |  |  |
| ≥60 vs <60 years | 0.26 (0.07-0.94) | 0.041 | | 0.28 (0.06-1.14) | 0.077 |
| ECOG status 1 vs 0 | 0.04 (0.00-5168.49) | 0.604 | |  |  |
| Distant metastasis site Yes vs No | |  | |  |  |
| Liver | 1.08 (0.23-4.94) | 0.914 | |  |  |
| Peritoneum | 3.35 (1.11-10.08) | 0.031 | | 4.27 (0.99-18.41) | 0.051 |
| Lymph nodes | 0.66 (0.22-2.00) | 0.472 | |  |  |
| Ovary | 3.96 (1.06-14.79) | 0.040 | | 0.23 (0.30-1.90) | 0.176 |
| Tumor size ≥5 vs <5 cm | 2.67 (0.82-8.69) | 0.103 | |  |  |
| Chemotherapeutic regimens Two-drug vs Three-drug | 1.06 (0.34-3.30) | 0.914 | |  |  |
| R0 Yes vs No | 0.27 (0.08-0.85) | 0.026 | | 0.31 (0.05-1.84) | 0.201 |
| Gastrectomy type Total vs Proximal | 1.42 (0.47-4.24) | 0.528 | |  |  |
| Signet ring cell Yes vs No | 6.33 (2.06-19.37) | 0.001 | | 6.29 (1.56-25.36) | 0.010 |
| Pathological stage |  |  | |  |  |
| T0-2 vs T3-4 | 0.01 (0.00-1.58) | 0.078 | |  |  |
| N0 vs N1-3 | 0.59 (0.19-1.83) | 0.368 | |  |  |
| M0 vs M1 | 0.33 (0.09-1.22) | 0.099 | |  |  |
| pCR Yes vs No | 0.03 (0.00-11.53) | 0.256 | |  |  |
| TRG 0-1 vs 2-3 | 0.03 (0.00-5.61) | 0.189 | |  |  |
| Abbreviations: BMI, body mass index; ECOG, eastern cooperative oncology group; HER2, human epidermal growth factor receptor 2; pCR, pathologic complete response; TRG, tumor regression grade; HR, hazard ratio; Cl, confidence intervals. | | | | | |
