# Supplementary material for: Clinical outcomes of conversion surgery following immune checkpoint inhibitors and chemotherapy in stage IV gastric cancer
Source: Int J Surg. 2023 Sep 14;109(12):4162–72. doi: 10.1097/JS9.0000000000000738 (PMC10720795; doi:10.1097/JS9.0000000000000738)
Supplement: SUPPLEMENTARY MATERIAL [file js9-109-4162-s007.docx]

| **eTable 3.** Evaluation of independent risk factors for conversion surgery by univariate and multivariate logistic regression analysis | | | | | |
| --- | --- | --- | --- | --- | --- |
|  | Univariable analysis | |  | Multivariable analysis | |
| Clinicopathologic variable | OR (95% CI) | P value | | OR (95% CI) | P value |
| Female vs male | 0.53 (0.24-1.14) | 0.108 | |  |  |
| Age at diagnosis |  |  | |  |  |
| ≥60 vs <60 years | 0.50 (0.24-1.06) | 0.072 | |  |  |
| ECOG status 1 vs 0 | 1.35 (0.13-13.38) | 0.797 | |  |  |
| TNM stage IV B vs IV A* | 5.65 (2.35-13.60) | <0.001 | |  |  |
| Distant metastasis site, Yes vs No |  |  | |  |  |
| Liver | 3.44 (1.11-10.62) | 0.032 | | 2.45 (0.70-8.54) | 0.159 |
| Peritoneum | 5.89 (2.64-13.14) | <0.001 | | 6.73 (2.61-17.37) | <0.001 |
| Lymph nodes | 4.34 (1.97-9.54) | <0.001 | | 4.50 (1.73-11.71) | 0.002 |
| Ovary | 1.54 (0.40-5.94) | 0.525 | |  |  |
| Tumor size ≥5 vs <5 cm | 1.47 (0.70-3.06) | 0.299 | |  |  |
| Signet ring cell Yes vs No | 1.80 (0.76-4.23) | 0.175 | |  |  |
| PD-L1 CPS≥5 Yes vs No | 0.31 (0.14-0.68) | 0.004 | | 0.22 (0.08-0.57) | 0.002 |
| HER2 positive Yes vs No | 0.23 (0.08-0.62) | 0.004 | | 0.31 (0.09-1.01) | 0.053 |
| CEA ≥5 vs <5 ng/ml | 1.59 (0.71-3.57) | 0.255 | |  |  |
| CA-199 ≥37 vs <37 U/ml | 2.34 (0.93-5.88) | 0.070 | |  |  |
| CA-724 ≥6 vs <6 U/ml | 1.99 (0.89-4.45) | 0.090 | |  |  |
| Abbreviations: ECOG, eastern cooperative oncology group; PD-L1, programmed cell death ligand 1;CPS, combined positive score; HER2, human epidermal growth factor receptor 2; MMR, mismatch repair; dMMR, proficient mismatch repair; pMMR, deficient mismatch repair; EBER, epstein-barr virus; CEA, carcinoembryonic antigen; CA-199, carbohydrate antigen199; CA-724, carbohydrate antigen 72-4; OR, odds ratio; Cl, confidence intervals. * Exclude multivariate analysis for strong correlation with distant metastasis site. | | | | | |
|  | | | | | |
